# Development and dosimetry of ^203^Pb/^212^Pb-labelled PSMA ligands: bringing “the lead” into PSMA-targeted alpha therapy?

**DOI:** 10.1007/s00259-018-4220-z

**Published:** 2019-01-03

**Authors:** José Carlos dos Santos, Martin Schäfer, Ulrike Bauder-Wüst, Wencke Lehnert, Karin Leotta, Alfred Morgenstern, Klaus Kopka, Uwe Haberkorn, Walter Mier, Clemens Kratochwil

**Affiliations:** 1 Department of Nuclear Medicine, University Hospital Heidelberg, INF 400, 69120 Heidelberg, Germany; 2 Division of Radiopharmaceutical Chemistry, German Cancer Research Center (DKFZ), Heidelberg, Germany; 3 ABX-CRO, Dresden, Germany; 4 Cooperation Unit Nuclear Medicine, German Cancer Research Center (DKFZ), Heidelberg, Germany; 5 Directorate for Nuclear Safety and Security, European Commission – Joint Research Centre, Karlsruhe, Germany; 6 German Cancer Consortium (DKTK), Heidelberg, Germany

**Keywords:** PSMA-targeted alpha therapy, ^212^Pb-CA012, Dosimetry, Prostate cancer

## Abstract

**Purpose:**

The aims of this study were to develop a prostate-specific membrane antigen (PSMA) ligand for labelling with different radioisotopes of lead and to obtain an approximation of the dosimetry of a simulated ^212^Pb-based alpha therapy using its ^203^Pb imaging analogue.

**Methods:**

Four novel Glu-urea-based ligands containing the chelators *p*-SCN-Bn-TCMC or DO3AM were synthesized. Affinity and PSMA-specific internalization were studied in C4-2 cells, and biodistribution in C4-2 tumour-bearing mice. The most promising compound, ^203^Pb-CA012, was transferred to clinical use. Two patients underwent planar scintigraphy scans at 0.4, 4, 18, 28 and 42 h after injection, together with urine and blood sampling. The time–activity curves of source organs were extrapolated from ^203^Pb to ^212^Pb and the calculated residence times of ^212^Pb were forwarded to its unstable daughter nuclides. QDOSE and OLINDA were used for dosimetry calculations.

**Results:**

In vitro, all ligands showed low nanomolar binding affinities for PSMA. CA09 and CA012 additionally showed specific ligand-induced internalization of 27.4 ± 2.4 and 15.6 ± 2.1 %ID/10^6^ cells, respectively. The ^203^Pb-labelled PSMA ligands were stable in serum for 72 h. In vivo, CA012 showed higher specific uptake in tumours than in other organs, and particularly showed rapid kidney clearance from 5.1 ± 2.5%ID/g at 1 h after injection to 0.9 ± 0.1%ID/g at 24 h. In patients, the estimated effective dose from 250–300 MBq of diagnostic ^203^Pb-CA012 was 6–7 mSv. Assuming instant decay of daughter nuclides, the equivalent doses projected from a therapeutic activity of 100 MBq of ^212^Pb-CA012 were 0.6 Sv_RBE5_ to the red marrow, 4.3 Sv_RBE5_ to the salivary glands, 4.9 Sv_RBE5_ to the kidneys, 0.7 Sv_RBE5_ to the liver and 0.2 Sv_RBE5_ to other organs; representative tumour lesions averaged 13.2 Sv_RBE5_ (where RBE5 is relative biological effectiveness factor 5). Compared to clinical experience with ^213^Bi-PSMA-617 and ^225^Ac-PSMA-617, the projected maximum tolerable dose was about 150 MBq per cycle.

**Conclusion:**

^212^Pb-CA012 is a promising candidate for PSMA-targeted alpha therapy of prostate cancer. The dosimetry estimate for radiopharmaceuticals decaying with the release of unstable daughter nuclides has some inherent limitations, thus clinical translation should be done cautiously.

**Electronic supplementary material:**

The online version of this article (10.1007/s00259-018-4220-z) contains supplementary material, which is available to authorized users.

## Introduction

Alpha radiation therapy with the “bone-seeker” ^223^RaCl_2_ has been shown to result in a survival benefit in patients with bone metastatic prostate cancer, but its beta-emitting analogue ^89^SrCl_2_ shows no such effect [1]. Well in line with these findings, two recent studies have shown higher response rates to ^225^Ac-PSMA-617 than to ^177^Lu-PSMA-617 [2, 3]. It has already been demonstrated in patients with neuroendocrine tumours that alpha-emitting ^213^Bi-DOTATOC can overcome resistance to beta-emitting ^90^Y/^177^Lu-DOTATOC [4]. Thus, interest in alpha emitter-based radionuclide therapy is increasing. However, there are only a few alpha emitters with an appropriate half-life between hours and days suitable for routine clinical use.

Despite being interesting for research, the short physical half-lives of ^213^Bi (0.8 h), ^212^Bi (1.0 h), ^149^Tb ( 4.1 h) and ^211^At (7.2 h) make their possible clinical application challenging. On the other hand, alpha emitters with long half-lives such as ^227^Th (18.7 days), that may be necessary to cope with the slow pharmacokinetics of full-length antibodies, may accumulate in the environment and may be associated with problems related to waste disposal if used on a large scale, e.g. for the treatment of epidemiologically important tumours. We consider that a physical half-life in the range 10 h to 10 days is favourable for clinical routine; this range includes ^212^Pb (*t*_½_ 10.6 h) and ^225^Ac (*t*_½_ 9.9 days). Therefore, we were interested in determining whether ^212^Pb might be a possible alternative in addition to ^225^Ac for PSMA-targeted alpha therapy (PSMA-TαT), a field that is becoming increasingly important.

Fortunately, the pharmacokinetic data needed to estimate the dosimetry of ^212^Pb-labelled radiopharmaceuticals can be acquired using ^203^Pb, which decays by emission of 279 keV gamma rays (80% abundance) that can be imaged in a conventional Anger camera and has a sufficient half-life (52 h) to cover four half-lives of its therapeutic analogue (^212^Pb; *t*_½_ 10.6 h).

Both 1,4,7,10-tetraazacyclododecane-1,4,7,10-tetraacetic acid (DOTA) and 1,4,7,10-tetra-(2-carbamoyl-methyl)-cyclododecane (TCMC) are known to form stable complexes with lead(II), TCMC at pH >2, DOTA at pH >3.5 [5–7]. Thus, regarding the physiologically relevant range (i.e. endosomes pH 4.5–6.5, blood pH 7.4), both chelators seem appropriate. DOTA-containing PSMA ligands can also form stable complexes with +3 metals at pH >1–2 [6], and several have already been evaluated [8]. In contrast, TCMC is more specific for Pb^2+^ and has not yet been systematically evaluated in the context of PSMA ligands. However, it has been reported that hydrophobic entities at the chelator or linker moiety can mediate the internalization efficiency of PSMA ligands [9], arousing interest in the evaluation of a new group of PSMA ligands that exploit the lower polarity of TCMC. This chelator could be conjugated to the root structure of PSMA-617 either via an SCN linker or using one of its four coordination arms.

The aim of this work was to develop PSMA ligands containing the chelators *p*-SCN-Bn-TCMC or DO3AM for labelling with different radioisotopes of lead. Using gamma-emitting ^203^Pb as an imaging surrogate, the dosimetry of a hypothetical ^212^Pb-PSMA-TαT ligand (using our most promising preclinical candidate) was projected.

## Materials and methods

All solvents and chemicals were purchased from Sigma Aldrich, Merck KGaA, Iris Biotech, or CheMatech, and used without further purification.

### Synthesis of PSMA ligands

**Fig. 1:**
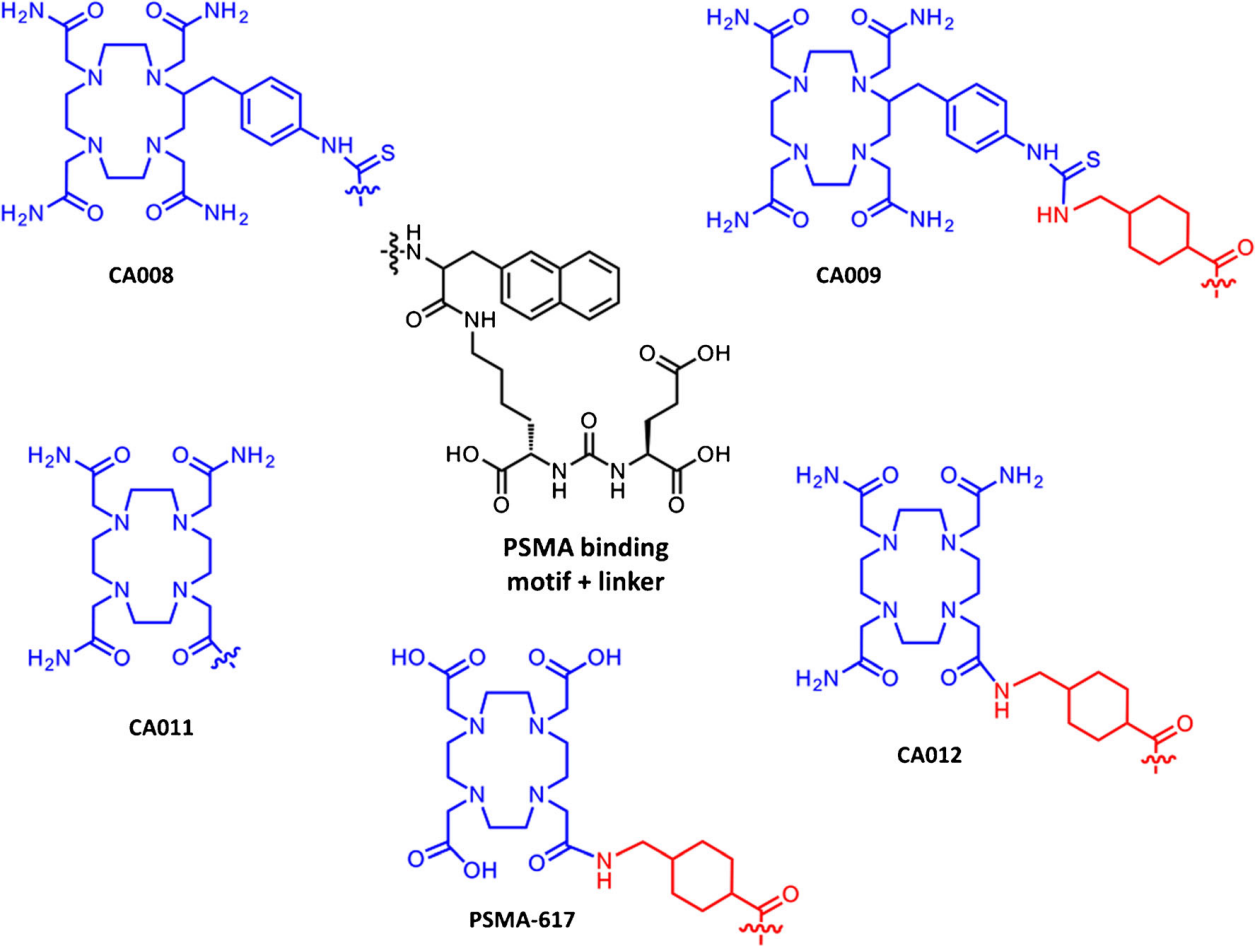
Chemical structures of the novel PSMA ligands using PSMA-617 as the lead structure

The first step of the solid-phase synthesis was the formation of the PSMA-binding motif on 2-chlorotrityl resin. For this purpose the isocyanate of the glutamyl moiety was formed using triphosgene and reacted with resin-immobilized ɛ-allyloxycarbonyl-protected lysine for 16 h. The allyloxycarbonyl-protecting group of the resulting urea binding motif was then cleaved. The chelators *p*-SCN-Bn-TCMC and DO3AM were attached directly or after coupling of the Fmoc-2-naphthylalanine, or conjugated after the linker *trans*-4-(Fmoc-aminomethyl)-cyclohexanecarboxylic acid. The compounds (Fig. 1) were cleaved from the resin and analysed by high-performance liquid chromatography (HPLC) and liquid chromatography–mass spectrometry (LC-MS). The crude products were purified by preparative HPLC on a Chromolith® SemiPrep column using gradient elution from 0.1% trifluoroacetic acid (TFA) in water to 0.1% TFA in acetonitrile. The final products were lyophilized and analysed by analytical HPLC (0–100% acetonitrile in water containing 0.1% TFA) on a 100 × 3 mm Chromolith Performance RP-18e column and LC-MS.

The conjugates (1 mM in water, 80 μl, 80 nmol) were added to 400 μl sodium acetate buffer (0.4 M in water, pH 5.0), 10 μl ascorbic acid (20% in water) and 140 μl ^203^Pb-chloride solution in 0.04 M HCl, with specific activity approximately 102.6 TBq/g (Lantheus Medical Imaging, North Billerica, MA, USA). The mixture was then heated at 95 °C for 5 min. Labelling was controlled by radio-HPLC. The stability of the ^203^Pb-labelled compounds was evaluated in 300 μl human serum at 37 °C for up to 72 h. After precipitation of the serum proteins by addition of two parts acetonitrile, the solution was centrifuged for 5 min at 13,000 rpm (twice) and the supernatant was analysed by radio-HPLC (0–100% MeCN over 5 min, monolith column). The data from the analysis of the conjugates are presented in Supplementary Table 1.

### Preclinical evaluation

In vitro and in vivo experiments were performed using the PSMA-positive C4-2 cell line, a subline of the LNCaP (lymph node carcinoma of the prostate) cell line (CRL-3314; American Type Culture Collection). C4-2 cells were cultivated in RPMI-1640 (PAN Biotech) supplemented with 10% fetal calf serum and stable glutamine (PAN Biotech). Cells were grown at 37 °C and incubated in humidified air equilibrated with 5% CO_2_.

### Competitive binding assay

A MultiScreen_HTS_-DV filter plate was incubated at room temperature with 100 μl phosphate-buffered saline (PBS) containing 1% bovine serum albumin (BSA) per well for 30 min. After removal of the PBS/BSA solution, 1 × 10^5^ C4-2 cells in Opti-MEM were added to each well. All nonlabelled compounds were dissolved in 300 μl Opti-MEM at the following concentrations: 0, 0.5, 1, 2.5, 5, 10, 25, 50, 100, 500, 1,000 and 5,000 nM. Subsequently, 3 μl of the radiolabelled compound was added, and 50 μl of this mixture was taken to obtain a solution containing 0.75 nM of the radiolabelled ligand. After 45 min incubation at 37 °C, the cells were washed twice with PBS, the radioactivity accumulated in the cells was collected, and the remaining radioactivity was measured in a gamma counter. The inhibitory potency was determined using ^68^Ga-labelled PSMA-HBED-CC dimer (i.e. PSMA-10) as the reference. The 50% inhibitory concentration was calculated using a nonlinear regression algorithm (GraphPad Prism 5.01 software). The experiments were performed in quadruplicate.

### Determination of internalization ratio

For determination of the specific internalization ratio, 24-well plates were incubated for 20 min with 0.1% poly-l-lysine in PBS at room temperature and washed once with PBS. In the next step, 1 ml RPMI containing 1 × 10^5^ C4-2 cells was added to each well and incubated overnight. The conditions during the experiment for each compound were: incubation at 37 °C or 4 °C with or without receptor blocking via 2-(phosphonomethyl)pentanedioic acid (2-PMPA; Axxora) at a final concentration of 500 μM. The cells were then incubated with 250 μl of a 30 nM solution of ^203^Pb-labelled compound. The plates were incubated either for 45 min in a water bath at 37 °C or on ice at 4 °C. The cells were then washed three times with 1 ml ice-cold PBS and incubated with 0.5 ml glycine (50 mM in HCl, pH 2.8) for 5 min. After an additional washing step with 1 ml ice-cold PBS, the cells were lysed with 0.5 ml 0.3 M NaOH and collected, and the radioactivity was measured with a gamma counter for 1 min. The specific cellular uptake was determined as the percentage of initially added radioactivity bound per 10^6^ cells (%IA/10^6^ cells) by subtraction of the respective uptake under blocking conditions. All experiments were performed in triplicate.

### Scintigraphic imaging and biodistribution

The in vivo experiments were carried out in accordance with the laws on animal welfare of the Federal Republic of Germany. For imaging and biodistribution studies, male BALB/c nu/nu mice (20–25 g; Charles River) bearing C4-2 tumour xenografts were used. The mice were inoculated subcutaneously in the right trunk with 5 × 10^6^ C4-2 cells in 50% Matrigel in Opti-MEM (1×). For small-animal imaging the mice were anaesthetized with 2% sevoflurane, and then 0.1 nmol (1.0 MBq) of the respective ^203^Pb ligand was injected into a tail vein. Serial planar scans were performed using a Gamma Imager-sct (Biospace Lab, Paris, France) with a parallel collimator (35 mm/1.8 mm/0.2 mm) after 10 min, 1 h, 4 h and 24 h. Based on the imaging results, compound CA012 was chosen for biodistribution studies. Experiments were performed in triplicate. For biodistribution, 0.025 nmol (1 MBq) of labelled compound per mouse was administered by injection into a tail vein. After 10 min, 1 h, 4 h and 24 h the mice were killed, the organs were dissected and weighed, and activity was measured using a gamma counter (Packard Cobra Auto-gamma). The organ uptakes were calculated as the percentage of the injected dose per gram of tissue (%ID/g).

### ^203^Pb-CA012 dosimetry and extrapolation to ^212^Pb-CA012

Two patients with castration-resistant metastasized prostate cancer referred to our department for planning of PSMA-targeted radioligand therapy underwent planar whole-body scans (GE Hawkeye Millennium; 1″ crystal, ME collimator, 279 keV peak ±10%, 8 cm/min) at 0.4, 4, 18, 28 and 42 h after injection of 258 and 310 MBq ^203^Pb-CA012, respectively. Images were loaded into the QDOSE dosimetry software suite (ABX-CRO, Dresden) and coregistered. Kidneys, liver, spleen, urinary bladder, salivary glands (both left and right parotid and submandibular glands) and several tumour lesions as well as a total-body region of interest (ROI) were segmented using the organ-dependent percentages of the maximum thresholds (15–65%) at the most suitable time-point and propagated to all other time-points performing an additional organ-based automatic rigid coregistration step. These ROIs were used to determine time–activity curves (TAC) for each organ, tumours and the total body. The first time-point (before voiding) of the uncorrected geometric mean images was used to calibrate the ROI counts in relation to the injected activity. The red marrow TAC was calculated from venous blood (six samples per patient) using established model assumptions [10, 11].

All TACs derived from the ^203^Pb data were recalculated using the replacement nuclide function of QDOSE, which automatically corrects all time-points for the physical decay of the source isotope, leaving only its biological clearance, and then the physical decay of the replacement radionuclide is applied. Biexponential curve fitting was applied to all organ TACs (with the exception of a few tumours and glands which had to be fitted monoexponentially). The accumulated activity was integrated assuming a linear increase from time 0 to the first measured time-point, numerically from the first to the last measured time-point using trapezoidal approximations and from the last measured time-point to infinity using the fit function. The activity in the remainder of the body was calculated by subtracting the activity of all source organs from that of the total body.

The residence times in the kidneys, liver, spleen, urinary bladder contents, red marrow and remainder of the body were exported for dose calculations in OLINDA 1.1, using the organ masses of the male adult phantom [12].

**Fig. 2:**
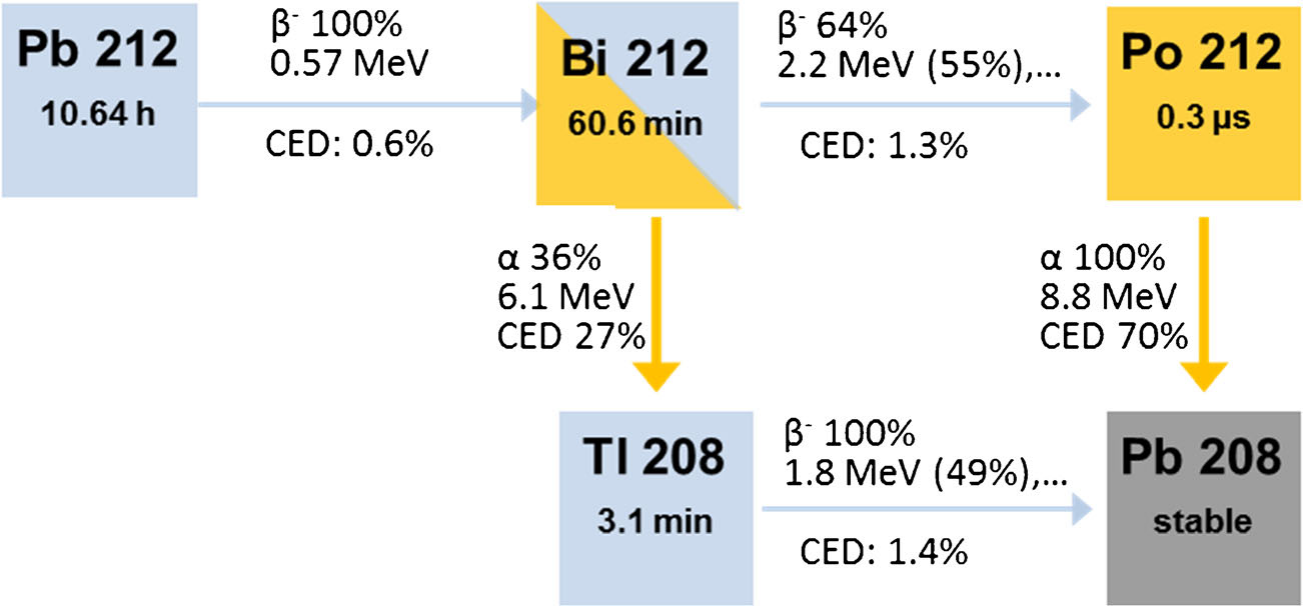
Decay scheme of ^212^Pb. CED percentage contribution to equivalent dose (assuming a relative biological effectiveness of 5 for alpha decay and 1 for gamma and beta decay)

The potential therapeutic nuclide ^212^Pb decays further to ^212^Bi, ^212^Po and ^208^Tl (Fig. 2). Assuming that the daughter nuclides remain at the site of decay of the parent nuclide and that there is a transient equilibrium between ^212^Pb and its daughters, the same residence times as for ^212^Pb were applied to the daughter nuclides. OLINDA calculations were performed for all nuclides individually. The respective decay steps were summed using weighting factors of 64.07% for ^212^Po and 35.93% for ^208^Tl according to their branching ratios (Fig. 2). According to the suggestions of the Committee on Medical Internal Radiation Dose and the US Department of Energy [13, 14], physical absorbed doses were translated into equivalent doses using weighting factors of 5 for alpha radiation and 1 for beta and photon radiation. thus reflecting the relative biological efficacy in regard to deterministic radiation effects – considered the leading factor in therapeutic settings. Tumour and salivary gland volumes were measured individually based on CT segmentation and their absorbed doses were approximated using a power function interpolating the spherical model estimates [15].

## Results

### Preclinical data

**Fig. 3:**
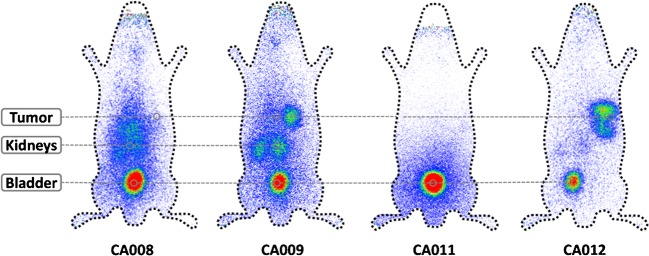
Planar scintigraphic images of ^203^Pb-labelled compounds in C4-2 tumour-bearing mice 1 h after injection via a tail vein

**Fig. 4:**
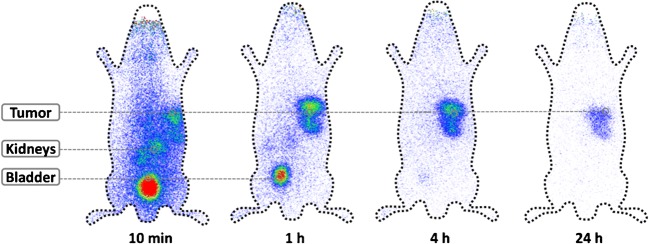
Time course of the distribution of ^203^Pb-CA012 in a C4-2 tumour-bearing mouse

The chemical structures of the four novel PSMA ligands together with that of PSMA-617 are presented in Fig. 1. All ligands, in particular CA011 and CA012 which have lost one coordination arm in comparison to original TCMC, showed high stability in human serum (up to 72 h checked). The affinities for the binding of PSMA to C4-2 cells (*K*i) were in the low two-digit nanomolar range for all ligands (Table 1). Despite their similar binding affinities, and probably due to either poor internalization or insufficient metabolic stability, CA008 and CA011 showed low tumour uptake in mice (Fig. 3). In contrast, ^203^Pb-CA009 and ^203^Pb-CA012 showed internalization rates of up to 27.4 ± 2.4%IA and 15.6 ± 2.1%IA per 10^6^ C4-2 cells (*n* = 3; Table 1). CA012 showed faster kidney clearance than CA009 and was therefore chosen for clinical translation (Fig. 4). In mice, ^203^Pb-PSMA-CA012 showed high tumour uptake of 8.4 ± 3.7%ID/g at 1 h after injection; the other biodistribution data in mice are presented in Supplementary Table 2.

**Table 1:** PSMA inhibition potencies (expressed as inhibition constants, *K*i) and specific Internalization (shown as lysate activity) values

Compound	*Ki* (nM)	Specific cell surface (%IA/10^6^ cells)	Specific lysate (%IA/10^6^ cells)
CA008	28.8 ± 3.4	n.d.	n.d.
CA009	15.3 ± 3.1	106.5 ± 2.8	27.4 ± 2.4
CA011	28.1 ± 2.9	n.d.	n.d.
CA012	24.3 ± 3.7	132.1 ± 4.3	15.6 ± 2.1

The data presented are means ± SD (*n* = 3)

*n.d.* not determined

### ^203^Pb imaging data in patients

**Fig. 5:**
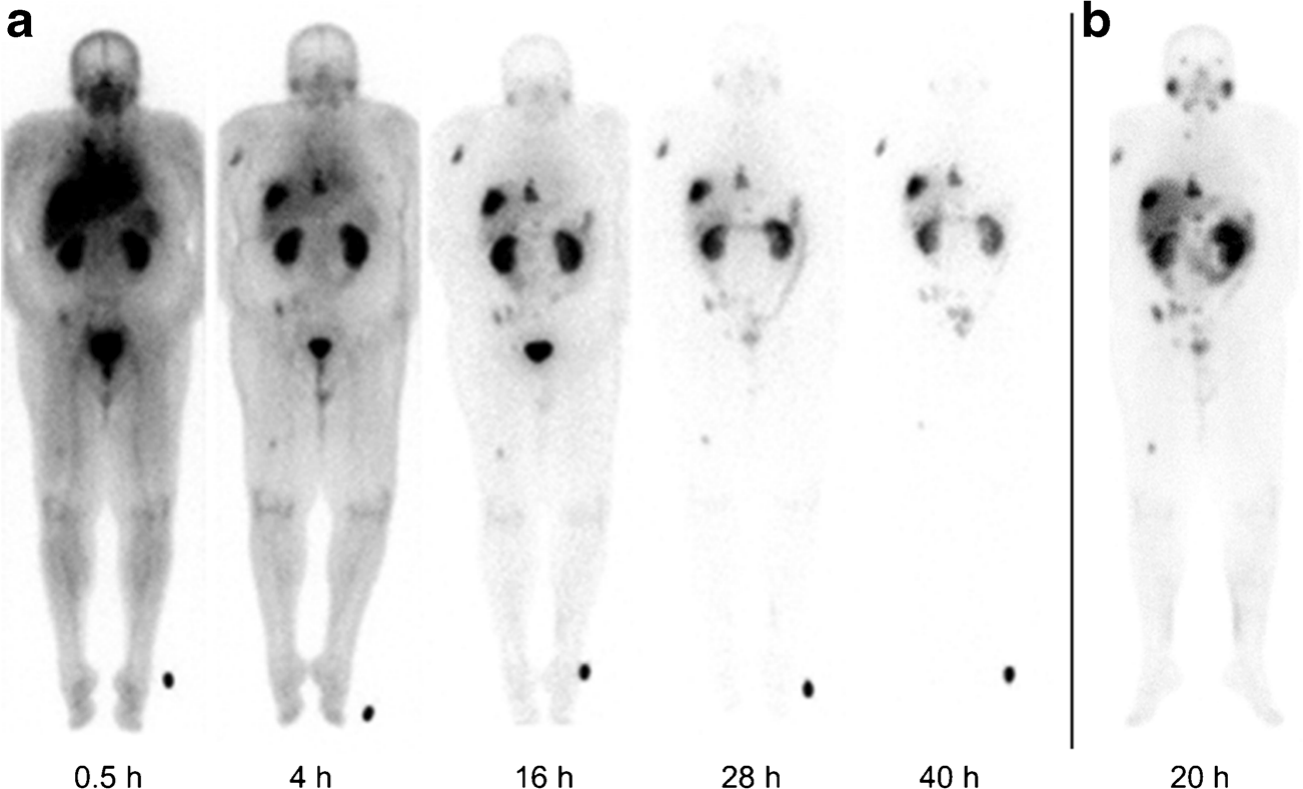
Geometric mean images of (**a**) ^203^Pb-CA012 planar scans over time and (**b**) a ^177^Lu-PSMA 617 treatment scan, all acquired with a medium-energy collimator

With an injected activity of 258–310 MBq, the 279 keV gamma rays emitted from ^203^Pb with an 80% abundance probability were sufficient for planar scans (Fig. 5a), but in comparison to a therapeutic approach using ^177^Lu-PSMA 617 with 7,400 MBq and emission of 210 keV gamma rays with 11% abundance probability (Fig. 5b), the count rate was three times lower and patients could not tolerate the time usually needed for single photon emission computed tomography (SPECT).

### Dosimetry estimates

The dosimetry estimates for diagnostic ^203^Pb-CA012 are shown in Table 2. All organ absorbed doses are dominated by photons (primary emission at 279 keV), and low probability emissions in sum contribute <10% in all organs. Clinical examinations with 250–300 MBq thus equal a radiation burden of 6.0–7.5 mSv. During decay from ^212^Pb to stable ^208^Pb, regardless of whether by the polonium or the thallium branch, two beta particles and one alpha particle are emitted per atom (Fig. 2). The extrapolated safety dosimetry estimate for therapeutic ^212^Pb-CA012, taking into account the complete succeeding decay chain, is also shown in Table 2. Assuming a relative biological effectiveness (RBE) of 5 for alpha and 1 for beta and gamma radiation, the equivalent doses for therapeutic ^212^Pb-CA012 consist of 96.4% alpha radiation, 2.2% beta radiation and 1.4% gamma radiation. Remarkably, the initial beta decay of ^212^Pb, which is directly traced by that of ^203^Pb, contributes less than 1% to the total equivalent dose; 99% of the equivalent dose arises from the succeeding daughter nuclides. The percentages of the total equivalent dose that are transferred during the respective decay steps are shown in Fig. 2 as the “CED” (contribution to equivalent dose) values.

**Table 2:** Safety dosimetry estimates for diagnostic ^203^Pb-CA012 and therapeutic ^212^Pb-CA012 based on the male adult phantom in OLINDA

Organ	^203^Pb-CA012		^212^Pb-CA012	
	Equivalent dose (mSv/100 MBq)	Standard deviation (mSv)	Equivalent dose (mSv _RBE5_/100 Mbq)	Standard deviation (mSv)
Adrenals	2.8	0.1	193.1	6.3
Brain	1.2	0.1	190.0	6.5
Breasts	1.1	0.1	190.0	6.4
Gallbladder	2.8	0.2	192.9	6.0
Lower large intestine	2.0	0.2	191.3	6.6
Small intestine	2.1	0.1	191.8	6.5
Stomach	2.0	0.1	191.8	6.5
Upper large intestine	2.1	0.1	191.8	6.5
Heart	1.8	0.1	191.3	6.4
Kidneys	18.1	0.8	4906.6	194.5
Liver	4.3	1.3	703.3	300.4
Lungs	1.6	0.1	190.9	6.4
Muscle	1.5	0.1	190.7	6.5
Pancreas	2.8	0.0	192.9	6.3
Red marrow	1.8	0.1	615.1	115.6
Osteogenic cells	3.7	0.3	3796.4	573.4
Skin	1.0	0.1	189.8	6.5
Spleen	6.7	0.4	1606.7	206.8
Testes	1.4	0.2	190.4	6.6
Thymus	1.4	0.1	190.7	6.5
Thyroid	1.4	0.2	190.5	6.6
Urinary bladder	6.7	0.8	200.2	8.6
Total body	1.7	0.1	237.8	0.7
Effective dose equivalent	3.6	0.1	748.9	17.2
Effective dose	2.4	0.3	415.1	13.5

For the diagnostic nuclide ^203^Pb, the equivalent dose is reported in sieverts because statistical radiation effects are the main interest at low absolute doses. For the therapeutic nuclide ^212^Pb, the equivalent dose is reported using a factor of 5 for the proprietary unit relative biological efficacy (RBE5) for alpha radiation to be predictive of deterministic radiation effects.

Amongst the OLINDA organs, the kidneys and red marrow might be dose limiting, together with the salivary glands which were assessed using the spherical model. The therapeutic range of a radiopharmaceutical is defined by the ratio between tumour dose and the dose to dose-limiting organs. The most relevant dosimetry information is summarized and compared to that of other PSMA-targeted alpha therapies [2, 16] in Table 3.

**Table 3:** Comparison of the dosimetry of ^212^Pb-CA012, ^13^Bi-PSMA-617 and ^225^Ac-PSMA-617 for the salivary glands, randomly chosen tumour lesions (sphere model) and the presumed dose-limiting organs

	^213^Bi-PSMA-617	^212^Pb-CA012	^225^Ac-PSMA-617
Nuclide half-life (h)	0.8	10.4	238
Projected treatment activity	1 GBq	100 MBq	6–8 MBq
Reference	[16]	This work	[2]
Tissue equivalent dose (Sv_RBE5_)^a^			
Salivary glands	8.1	7.5	13.8
Kidneys	8.1	4.9	4.2
Red marrow	0.5	0.6	0.3
Tumour (mean)	7.6	14	34
Tumour/salivary glands	0.9	1.9	2.5
Tumour/kidneys	0.9	2.9	8.1
Tumour/red marrow	15.2	23.3	113.3

^a^The equivalent dose is reported using a factor of 5 for the proprietary unit relative biological efficacy (RBE5) for alpha radiation .

## Discussion

The aim of this work was to develop PSMA ligands containing the chelators *p*-SCN-Bn-TCMC or DO3AM suitable for labelling with different isotopes of lead. Except the chelator, the ligands follow the lead structure of PSMA-617 [8] which belongs to the class Glu-ureido PSMA inhibitors bearing the 2-naphthylalanine and cyclohexanecarboxylic acid linker, to target the catalytic domain of PSMA [17] and to interact favourably with its lipophilic accessory pocket [18] and arene-binding site [19]. The development of PSMA-617 and its favourable interaction with the PSMA protein crystal has recently been reviewed [20, 21]. However, the optimal hydrophilicity of the chelator region has not yet been systematically elaborated.

In vitro all new ligands show low two-digit nanomolar affinity, slightly inferior to the single-digit affinity of the reference compound PSMA-617 [8]. Nevertheless, comparison of PSMA-10 and PSMA-11 has already demonstrated that (once a sufficient level has been reached) moderate differences in affinity alone are not sufficient to predict the respective in vivo performance of one particular high-affinity ligand [22]. Another important parameter for the therapeutic appropriateness of a ligand, especially with a radiolabel that decays through unstable daughter nuclides, is internalization. ^203^Pb-CA009 and ^203^Pb-CA012 were specifically internalized with uptake values of approximately 27%ID and 16%ID per 10^6^cells, comparable to the 18%ID per 10^6^cells that we have previously determined for ^177^Lu-PSMA-617 [8].

In mice, ^203^Pb-PSMA-CA012 was found to be the most promising of our candidates and showed a high tumour uptake of 8.4 ± 3.7%ID/g at 1 h after injection, which is nearly identical to the tumour uptake of 8.5 ± 4.1%ID/g reported for ^68^Ga-PSMA-617 [8]. The early kidney accumulation of CA012 at 1 h after injection was markedly lower (5.1 ± 2.5%ID/g) than that of PSMA-617 (113.3 ± 24.4%ID/g); however, this advantage was less after 24 h (CA012 2%, PSMA-617 1%). Thus, regarding a possible clinical benefit, the dedicated combination of CA012 and a short half-life nuclide such as ^212^Pb is most promising.

In humans, the accuracy of our dosimetry estimate was limited by the low count rate that could be achieved with the injected 250–300 MBq ^203^Pb-CA012. Consequently, we were only able to assess the activity distribution on planar scans and not on SPECT scans. It is reasonable that without any clinical data available in the first-in-human studies, the activities had to be chosen very cautiously. However, our preliminary (i.e. planar) dosimetry estimate, with an effective dose of 2.4 mSv per 100 MBq ^203^Pb-CA012, suggest that it would be reasonable to inject up to 750 MBq (approximately 18 mSv effective dose) in future dosimetry studies. These studies could then use the SPECT technique, which allows volume segmentation of activity and would also make the application of other techniques suggested for state-of-the-art dosimetry studies reasonable [23].

The extrapolation of the dosimetry from diagnostic ^203^Pb-CA012 to therapeutic ^212^Pb-CA012 also has limitations: The most relevant uncertainty of the model used is the simplification that the residence time of ^212^Pb-CA012 can be forwarded directly to all daughter nuclides, neglecting the possibility of interim translocation. As illustrated in Fig. 2, the initial beta decay of ^212^Pb-CA012 – only the location of this decay step is perfectly traced by the gamma emission from the imaging surrogate ^203^Pb-CA012 – contributes less than 1% to the summed dose of the whole ^212^Pb decay chain, which is dominated by the alpha emissions from the succeeding daughter nuclides. Although DOTA and TCMA form stable complexes with both lead(II) and bismuth(III) isotopes and the recoil beta emission is negligible in comparison to alpha emission, it has been reported that during the decay from ^212^Pb to ^212^Bi, 36% of the daughter nuclide can be lost from the chelator [24].

Therefore, it is mandatory that our projected dosimetry approximations are verified before moving forward from ^203^Pb to ^212^Pb in future studies. As the effect of losing daughter nuclides from the chelator complex and redistribution according to the inherent characteristics of these elements is most relevant during the extracellular circulation phase, organs from mice or blood samples from patients treated with ^212^Pb-CA012 should be characterized in vitro regarding the ratio of complexed lead and free bismuth. However, due to the rapid tumour targeting and fast internalization of low molecular weight PSMA ligands, the circulation time is much shorter than that of full-length antibodies evaluated for radioimmunotherapy [8, 25, 26].

Once internalized, precipitation with organic anions from cytosolic proteins may sufficiently decelerate the efflux of metal ions and even the 1-h half-life of ^212^Bi might be short enough to consider most (but presumably not all) of it “functionally trapped”. Due to the extremely short half-life of ^212^Po (0.3 μs) and the physiological intracellular accumulation of ^208^Tl (*t*_½_ 3.1 min; due to its rapid intracellular accumulation ^201^Tl is even used in perfusion scintigraphy [27]), it is reasonable to assume that translocation of the intracellularly generated succeeding daughter nuclides is of secondary relevance or even negligible. At this point it should be mentioned, that more than 50 patients have already been successfully treated with ^225^Ac-PSMA-617, even though ^225^Ac decays with four alpha and two beta decays, which make the possible translocation of its various daughter nuclides an even more critical issue in comparison to ^212^Pb [3, 28]. ^223^RaCl_2_, another radiopharmaceutical with several daughter nuclides, targets the extracellular bone matrix and the first decay product, ^219^Rn, is a freely diffusible inert gas with a half-life of 4.0 s. However, mobility and diffusion back to the systemic circulation were not relevant issues even for extracellularly generated decay products [29]. As even 1.11 MBq of free ^212^Pb could be injected into mice with body weights in the range 19–28 g without relevant toxicity on days 7 and 90 [30], there are remarkable safety margins regarding the in vivo stability of the lead complex and some redistribution of daughter radionuclides.

The clinical value of dosimetry modelling of therapeutic radiopharmaceuticals is that it provides an approximation of their maximum tolerable doses (MTD) that would allow empirical dose-escalation trials to be shortened or eventually even omitted. With the clinical introduction of alpha emitters, we are now facing new challenges because historically the tolerance limits of normal tissue reported in gray have been evaluated using beta and gamma radiation, and due to different RBEs of alpha emitters these thresholds are not valid if the physically exact unit for the absorbed dose (gray) is applied directly. Regarding statistical radiation effects a weighting factor of 20 is recommended for converting to the equivalent dose in sieverts. However, we want to predict antitumour activity and side effects, i.e. deterministic radiation effects, and no generally accepted nomenclature for this kind of equivalent dose is yet available.

As all alpha emitters considered clinically have different ratios of alpha, beta and gamma emissions, it does not make sense to report summed absorbed doses in gray, because it does not seem practicable to apply one weighting factor to a mixture of various, biologically different, kinds of radiation. Thus, we recently introduced a proprietary metric in which the absorbed doses of alpha, beta and gamma radiation are first multiplied by their most appropriate weighting factor and then summed. The correction factors used are provided as indices. However, we have to emphasize that the RBE is not a formal SI unit. Also the RBE factor of 5 for alpha radiation reflects only an average factor found in the literature [13, 14] and individual results can vary. Nevertheless, this concept has already been used to project the MTD of ^225^Ac-PSMA-617 [2] and ^213^Bi-PSMA-617 [16]. The results of empirical dose escalation accompanying the clinical translation of ^225^Ac-PSMA-617 [2] and the successful clinical application of ^213^Bi-PSMA-617 [31] without the need for amending empirical data have confirmed the practical usefulness of this approach.

In using the RBE5 metric to compare the dosimetry approximations of ^212^Pb-CA012 with those of ^213^Bi-PSMA-617 and ^225^Ac-PSMA-617 (Table 3), we assume that the MTD of ^212^Pb-CA012 would be approximately 150 MBq per cycle regarding acute haematological toxicity and xerostomia, while the cumulative dose to the kidneys, the dose-limiting organ, could be about 400–600 MBq. However, due to the methodological limitations discussed, these approximations are still preliminary, and we need more experience with other nuclide/tracer combinations and long-term follow-up regarding delayed toxicities to refine our model. Presently, an empirical phase 2 study still appears necessary to evaluate the dose range between 50 and 150 MBq for single doses of ^212^Pb-CA012. ^212^Pb-TCMC-trastuzumab is so far the only ^212^Pb-labelled radiopharmaceutical to have been evaluated in a formal clinical trial [32, 33], but it is administered intraperitoneally, and there is neither a strong demand for imaging-based dosimetry, nor can the development of a systemically administered radiopharmaceutical be guided by dose escalation of this local therapy. However, other small molecules, e.g. for therapy of melanoma [34, 35] and neuroendocrine tumours [36], are currently on the way to clinical translation, and due to their encouraging fast pharmacokinetics they are considered suitable candidates for labelling with ^212^Pb.

Comparing the potentially achievable tumour absorbed doses of ^212^Pb-CA012 with those of other alpha emitter-labelled PSMA ligands (Table 3), there seems to be a small advantage for ^225^Ac-PSMA-617. However, taking into account the limited precision of our planar dosimetry especially for small metastases, the random error related to the fact that data were obtained from only two patients, and the still limited knowledge about the potential impact of different dose rates, it is too early to draw a final conclusion.

In summary, due to the theoretically well scalable on-demand production of ^203^Pb in cyclotrons and of ^212^Pb from ^224^Ra generators [37] and after demonstration of automated cassette-based labelling procedures suitable for clinical use [38], the ^203^Pb/^212^Pb tandem approach is a reasonable option for overcoming the current supply limitation of appropriate medical alpha emitters.

### Conclusion

PSMA-TαT shows promise for the treatment of metastatic prostate cancer. The novel ligands, in particular CA012, are promising shuttles for labelling with ^212^Pb. Once ^212^Pb becomes more routinely available, clinical evaluation of ^212^Pb-CA012 is warranted. Due to the inherent methodological limitations in the dosimetry of radiopharmaceuticals decaying through unstable daughter nuclides, our approximations can only provide a level of guidance and clinical dose finding should still be amended by an empirical dose-escalation study.

## Electronic supplementary material

ESM 1 (DOCX 16 kb)
