# Supplementary material for: Development and dosimetry of 203Pb/212Pb-labelled PSMA ligands: bringing “the lead” into PSMA-targeted alpha therapy?
Source: Eur J Nucl Med Mol Imaging. 2019 Jan 3;46(5):1081–91. doi: 10.1007/s00259-018-4220-z (PMC6451745; doi:10.1007/s00259-018-4220-z)
Supplement: Supplementary file 1 — (DOCX 16 kb) [file 259_2018_4220_MOESM1_ESM.docx]

**Supplemental Data**

**Development and dosimetry of Pb-203 / Pb-212 labeled PSMA ligands – Bringing “the Lead” into PSMA-Targeting Alpha Therapy?**

**Supplemental Table 1**

Analytic Data of the Pb-Ligands

| Compound | m/z (calculated) | [^203^Pb-Ligand]-HPLC retention time (min)* | m/z (determined) ** |
| --- | --- | --- | --- |
| **CA008** | 1063.49 | 2.52 | 1064.49 |
| **CA009** | 1202.59 | 2.60 | 1203.59 |
| **CA011** | 899.45 | 2.16 | 900.45 |
| **CA012** | 1038.55 | 2.40 | 1039.55 |

*HPLC column Chromolith Performance RP-18e 100 x 3 mm, 0-100% acetonitrile in water + 0.1% trifluoroacetic acid; **Mass spectrometry of non-labeled ligands detected as [M+H]^+^

**Supplemental Table 2**

Organ distribution of ^203^Pb-PSMA-CA012 in tumor bearing mice

| Tissue | 10 min | 1 h | 4 h | 24 h | |
| --- | --- | --- | --- | --- | --- |
| Blood | 5.37 ± 1.95 | 0.26 ± 0.10 | 0.05 ± 0.01 | | 0.03 ± 0.00 |
| Heart | 1.78 ± 0.66 | 0.10 ± 0.03 | 0.04 ± 0.02 | | 0.02 ± 0.00 |
| Lung | 4.31 ± 1.19 | 0.33 ± 0.13 | 0.10 ±0.03 | | 0.05 ± 0.01 |
| Spleen | 1.97 ± 1.07 | 0.16 ± 0.08 | 0.07 ± 0.01 | | 0.07 ± 0.02 |
| Liver | 1.25 ± 0.43 | 0.20 ± 0.10 | 0.09 ± 0.01 | | 0.10 ± 0.02 |
| Kidneys | 26.97 ± 8.74 | 5.08 ± 2.45 | 1.61 ± 0.28 | | 0.89 ± 0.14 |
| Muscle | 1.20 ± 0.43 | 0.05 ± 0.03 | 0.06 ± 0.03 | | 0.02 ± 0.00 |
| Small intestine | 1.75 ± 0.48 | 0.10 ± 0.04 | 0.07 ± 0.05 | | 0.02 ± 0.00 |
| Brain | 0.14 ± 0.04 | 0.02 ± 0.00 | 0.02 ± 0.01 | | 0.01 ± 0.00 |
| Tumor | 12.33 ± 1.92 | 8.37 ± 3.67 | 7.81 ±0.86 | | 3.30 ± 1.60 |

Organ distribution of 0.025 nmol of ^203^Pb –PSMA CA012. Values are expressed in % ID/g of tissue ± standard deviation; n = 3 for all tissues.
